# Supplementary material for: Modeling glioblastoma heterogeneity as a dynamic network of cell states
Source: Mol Syst Biol. 2021 Sep 16;17(9):e10105. doi: 10.15252/msb.202010105 (PMC8444284; doi:10.15252/msb.202010105)
Supplement: Supplementary file 6 — Source Data for Figure 5 [file MSB-17-e10105-s004.zip › Figure5A_sourcedata/GSEA_3017/nosigdb_stateD.GseaPreranked.1622795010397/DOROSZKO_U3054MG_CLUSTER_8.html]

Details for gene set DOROSZKO\_U3054MG\_CLUSTER\_8[GSEA]

|  || Dataset | state23017 |
| Phenotype | NoPhenotypeAvailable |
| Upregulated in class | na\_pos |
| GeneSet | DOROSZKO\_U3054MG\_CLUSTER\_8 |
| Enrichment Score (ES) | 0.13674138 |
| Normalized Enrichment Score (NES) | 1.2997966 |
| Nominal p-value | 0.0 |
| FDR q-value | 0.37879625 |
| FWER p-Value | 0.74 |
Table: GSEA Results Summary

  

Fig 1: Enrichment plot: DOROSZKO\_U3054MG\_CLUSTER\_8      
 Profile of the Running ES Score & Positions of GeneSet Members on the Rank Ordered List

  

| PROBE | GENE SYMBOL | GENE\_TITLE | RANK IN GENE LIST | RANK METRIC SCORE | RUNNING ES | CORE ENRICHMENT || 1 | DDX21 |  |  | 5 | 0.579 | 0.0141 | Yes |
| 2 | SOX11 |  |  | 6 | 0.578 | 0.0322 | Yes |
| 3 | AC020916.1 |  |  | 21 | 0.451 | 0.0349 | Yes |
| 4 | IDI1 |  |  | 34 | 0.382 | 0.0371 | Yes |
| 5 | PURB |  |  | 41 | 0.370 | 0.0438 | Yes |
| 6 | AL118516.1 |  |  | 42 | 0.368 | 0.0553 | Yes |
| 7 | TRIO |  |  | 76 | 0.314 | 0.0382 | Yes |
| 8 | GDF11 |  |  | 97 | 0.294 | 0.0311 | Yes |
| 9 | MAP3K1 |  |  | 103 | 0.289 | 0.0361 | Yes |
| 10 | CHD7 |  |  | 116 | 0.278 | 0.0350 | Yes |
| 11 | AC012360.3 |  |  | 121 | 0.275 | 0.0403 | Yes |
| 12 | JMJD1C |  |  | 153 | 0.256 | 0.0231 | Yes |
| 13 | POU2F1 |  |  | 194 | -0.253 | -0.0016 | Yes |
| 14 | EIF4G3 |  |  | 205 | -0.254 | -0.0018 | Yes |
| 15 | STAT2 |  |  | 207 | -0.254 | 0.0053 | Yes |
| 16 | SREK1 |  |  | 212 | -0.255 | 0.0101 | Yes |
| 17 | TTC3 |  |  | 221 | -0.255 | 0.0115 | Yes |
| 18 | CROT |  |  | 224 | -0.256 | 0.0179 | Yes |
| 19 | RALGAPA1 |  |  | 230 | -0.257 | 0.0219 | Yes |
| 20 | NF1 |  |  | 254 | -0.260 | 0.0113 | Yes |
| 21 | UBR5 |  |  | 265 | -0.261 | 0.0113 | Yes |
| 22 | FUS |  |  | 290 | -0.263 | -0.0001 | Yes |
| 23 | MBNL2 |  |  | 292 | -0.263 | 0.0074 | Yes |
| 24 | TCTEX1D2 |  |  | 297 | -0.263 | 0.0123 | Yes |
| 25 | SNX13 |  |  | 301 | -0.264 | 0.0182 | Yes |
| 26 | PKP4 |  |  | 316 | -0.265 | 0.0150 | Yes |
| 27 | ZEB2 |  |  | 317 | -0.265 | 0.0233 | Yes |
| 28 | DOCK7 |  |  | 321 | -0.266 | 0.0292 | Yes |
| 29 | ARMC1 |  |  | 326 | -0.266 | 0.0343 | Yes |
| 30 | MYO9A |  |  | 356 | -0.268 | 0.0190 | Yes |
| 31 | USP33 |  |  | 375 | -0.271 | 0.0128 | Yes |
| 32 | RBMS1 |  |  | 377 | -0.271 | 0.0205 | Yes |
| 33 | ATXN2 |  |  | 389 | -0.272 | 0.0201 | Yes |
| 34 | ST5 |  |  | 392 | -0.272 | 0.0270 | Yes |
| 35 | NPDC1 |  |  | 402 | -0.274 | 0.0282 | Yes |
| 36 | RIF1 |  |  | 403 | -0.274 | 0.0368 | Yes |
| 37 | CDC42BPA |  |  | 415 | -0.275 | 0.0364 | Yes |
| 38 | RBM5 |  |  | 423 | -0.276 | 0.0394 | Yes |
| 39 | LMF2 |  |  | 425 | -0.276 | 0.0472 | Yes |
| 40 | S100A13 |  |  | 427 | -0.276 | 0.0550 | Yes |
| 41 | TUBGCP5 |  |  | 443 | -0.279 | 0.0515 | Yes |
| 42 | ARID4A |  |  | 448 | -0.279 | 0.0570 | Yes |
| 43 | CASP3 |  |  | 459 | -0.280 | 0.0577 | Yes |
| 44 | ATM |  |  | 460 | -0.280 | 0.0664 | Yes |
| 45 | BRD2 |  |  | 461 | -0.281 | 0.0752 | Yes |
| 46 | PEX2 |  |  | 468 | -0.282 | 0.0792 | Yes |
| 47 | IFRD1 |  |  | 469 | -0.282 | 0.0880 | Yes |
| 48 | ANKRD36C |  |  | 501 | -0.285 | 0.0716 | Yes |
| 49 | ATP2C1 |  |  | 505 | -0.286 | 0.0781 | Yes |
| 50 | NREP |  |  | 509 | -0.286 | 0.0846 | Yes |
| 51 | RNF213 |  |  | 533 | -0.289 | 0.0749 | Yes |
| 52 | RUFY3 |  |  | 539 | -0.290 | 0.0799 | Yes |
| 53 | DYRK1A |  |  | 551 | -0.291 | 0.0801 | Yes |
| 54 | PSPH |  |  | 582 | -0.296 | 0.0649 | Yes |
| 55 | EVI5 |  |  | 587 | -0.297 | 0.0709 | Yes |
| 56 | ACTN4 |  |  | 604 | -0.301 | 0.0673 | Yes |
| 57 | CCDC93 |  |  | 610 | -0.303 | 0.0727 | Yes |
| 58 | AC004951.1 |  |  | 619 | -0.304 | 0.0757 | Yes |
| 59 | NCOR2 |  |  | 628 | -0.306 | 0.0788 | Yes |
| 60 | SCAPER |  |  | 673 | -0.314 | 0.0527 | Yes |
| 61 | TRIM37 |  |  | 689 | -0.317 | 0.0504 | Yes |
| 62 | CCNL2 |  |  | 702 | -0.320 | 0.0506 | Yes |
| 63 | RAB10 |  |  | 708 | -0.321 | 0.0566 | Yes |
| 64 | GCC2 |  |  | 712 | -0.321 | 0.0642 | Yes |
| 65 | PILRB |  |  | 718 | -0.323 | 0.0702 | Yes |
| 66 | PTAR1 |  |  | 730 | -0.325 | 0.0715 | Yes |
| 67 | CPLANE1 |  |  | 762 | -0.335 | 0.0567 | Yes |
| 68 | PFKL |  |  | 771 | -0.337 | 0.0607 | Yes |
| 69 | GOLGB1 |  |  | 775 | -0.338 | 0.0688 | Yes |
| 70 | SFXN5 |  |  | 787 | -0.340 | 0.0705 | Yes |
| 71 | ZNF680 |  |  | 796 | -0.342 | 0.0747 | Yes |
| 72 | CEP192 |  |  | 802 | -0.345 | 0.0814 | Yes |
| 73 | KIAA1109 |  |  | 803 | -0.345 | 0.0922 | Yes |
| 74 | AHI1 |  |  | 806 | -0.347 | 0.1015 | Yes |
| 75 | GNB5 |  |  | 827 | -0.351 | 0.0962 | Yes |
| 76 | PCM1 |  |  | 834 | -0.353 | 0.1023 | Yes |
| 77 | MICAL3 |  |  | 840 | -0.355 | 0.1094 | Yes |
| 78 | ARFGEF1 |  |  | 845 | -0.356 | 0.1173 | Yes |
| 79 | CLSTN1 |  |  | 855 | -0.359 | 0.1212 | Yes |
| 80 | CFLAR |  |  | 873 | -0.364 | 0.1187 | Yes |
| 81 | SYNE1 |  |  | 877 | -0.365 | 0.1277 | Yes |
| 82 | C1QTNF6 |  |  | 887 | -0.368 | 0.1319 | Yes |
| 83 | DST |  |  | 913 | -0.379 | 0.1234 | Yes |
| 84 | VPS13A |  |  | 931 | -0.386 | 0.1216 | Yes |
| 85 | MACF1 |  |  | 940 | -0.390 | 0.1273 | Yes |
| 86 | AKAP9 |  |  | 959 | -0.398 | 0.1251 | Yes |
| 87 | WSB1 |  |  | 961 | -0.398 | 0.1367 | Yes |
| 88 | CCP110 |  |  | 1010 | -0.420 | 0.1107 | No |
| 89 | SRGAP2C |  |  | 1029 | -0.432 | 0.1096 | No |
| 90 | ITPR2 |  |  | 1053 | -0.447 | 0.1048 | No |
| 91 | KLHDC8A |  |  | 1059 | -0.451 | 0.1149 | No |
| 92 | ZBTB20 |  |  | 1061 | -0.451 | 0.1282 | No |
| 93 | GAS7 |  |  | 1085 | -0.462 | 0.1239 | No |
| 94 | SRGAP3 |  |  | 1188 | -0.567 | 0.0585 | No |
| 95 | NEAT1 |  |  | 1315 | -1.543 | 0.0041 | No |
Table: GSEA details [plain text format]

  

Fig 2: DOROSZKO\_U3054MG\_CLUSTER\_8: Random ES distribution      
 Gene set null distribution of ES for **DOROSZKO\_U3054MG\_CLUSTER\_8**
